# Microwave- and Ultrasound-Assisted Extraction of Cannabinoids and Terpenes from Cannabis Using Response Surface Methodology

**DOI:** 10.3390/molecules27248803

**Published:** 2022-12-12

**Authors:** Philip Wiredu Addo, Sai Uday Kumar Reddy Sagili, Samuel Eichhorn Bilodeau, Frederick-Alexandre Gladu-Gallant, Douglas A. MacKenzie, Jennifer Bates, Garnet McRae, Sarah MacPherson, Maxime Paris, Vijaya Raghavan, Valérie Orsat, Mark Lefsrud

**Affiliations:** 1 Bioresource Engineering Department, Macdonald Campus, McGill University, Ste-Anne-De-Bellevue, Montreal, QC H9X 3V9, Canada; 2 EXKA Inc., 7625 Route Arthur Sauvé, Mirabel, QC J7N 2R6, Canada; 3 National Research Council of Canada, Metrology, 1200 Montreal Road, Ottawa, ON K1A 0R6, Canada

**Keywords:** cannabis, cannabinoids, delta-9-tetrahydrocannabinol, extraction, microwave, ultrasound

## Abstract

Limited studies have explored different extraction techniques that improve cannabis extraction with scale-up potential. Ultrasound-assisted and microwave-assisted extraction were evaluated to maximize the yield and concentration of cannabinoids and terpenes. A central composite rotatable design was used to optimize independent factors (sample-to-solvent ratio, extraction time, extraction temperature, and duty cycle). The optimal conditions for ultrasound- and microwave-assisted extraction were the sample-to-solvent ratios of 1:15 and 1:14.4, respectively, for 30 min at 60 °C. Ultrasound-assisted extraction yielded 14.4% and 14.2% more oil and terpenes, respectively, compared with microwave-assisted extracts. Ultrasound-assisted extraction increased cannabinoid concentration from 13.2–39.2%. Considering reference ground samples, tetrahydrocannabinolic acid increased from 17.9 (g 100 g dry matter^−1^) to 28.5 and 20 with extraction efficiencies of 159.2% and 111.4% for ultrasound-assisted and microwave-assisted extraction, respectively. Principal component analyses indicate that the first two principal components accounted for 96.6% of the total variance (PC1 = 93.2% and PC2 = 3.4%) for ultrasound-assisted extraction and 92.4% of the total variance (PC1 = 85.4% and PC2 = 7%) for microwave-assisted extraction. Sample-to-solvent ratios significantly (*p* < 0.05) influenced the secondary metabolite profiles and yields for ultrasound-assisted extracts, but not microwave-assisted extracts.

## 1. Introduction

The extraction of secondary compounds from cannabis presents several challenges. Cannabinoids and terpenoids decompose with light and heat, making them unstable during sample preparation, extraction, and testing methods [1,2,3]. Additionally, differences in the quality and quantity of the extracted crude oil can be attributed to factors such as cannabis plant type (drug or fibre), pollination, sex, age, plant parts, method of plant cultivation (indoor or outdoor), harvest conditions, drying, and storage [4,5,6]. Extraction techniques for cannabis biomass have evolved quickly, resulting in diverse methodologies that have not been properly validated [7]. Frequently used techniques in industries for quality assurance and control include cold ethanol extraction, supercritical CO_2_ extraction, conventional Soxhlet extraction, ultrasound-assisted extraction, and microwave-assisted extraction [8,9]. Most researchers report that microwave-assisted extraction and ultrasound-assisted extraction are comparably efficient when compared with traditional solvent methods [10,11,12,13].

Microwaves are non-ionizing irradiation that excites molecules in the essential oil, thereby increasing the rate of extraction [14,15]. Microwaves may be used in conjunction with solvent extraction, Soxhlet extraction, and distillation [11,16,17,18]. Importantly, microwave-assisted extraction is a safe and environmentally friendly method, as it reduces solvent use and energy consumption, along with various environmental hazards such as chemical wastes. Research studies have concluded that the concentration of secondary metabolites in extracts can be increased using a microwave-assisted extraction system [19,20,21].

Compared with some other novel extraction techniques, the ultrasonic device is less expensive and is very easy to use [22]. Ultrasound-assisted extraction is a rapid, simple, and eco-friendly method for extracting bioactive metabolites from plants, with reduced initial production costs due to the low energy and process time required [23]. Ultrasound-assisted extraction uses acoustic cavitation to produce cavitation bubbles which implode and exert mechanical forces which improve the extraction process by increasing solvent penetration into the plant matrix [24]. Extraction rates are increased by the macroturbulence and high-velocity inter-particle collisions that are caused by the implosion of the gas bubbles [25,26]. Ethanol was used as the extraction solvent, as it is commonly used in the cannabis industry and considered a “green” solvent. Ultrasound and microwaves are considered improved extraction techniques compared with conventional systems, with several advantages, such as shortened extraction time, decreased solvent volumes, and increased extract yield [19,27,28,29]. However, both techniques have not been fully explored for cannabis extraction. 

The aim of this study was to determine and compare the optimal extraction conditions for cannabis using ultrasound-assisted extraction (UAE) and microwave-assisted extraction (MAE). Microwave- and ultrasound-assisted extraction were used for the study, as both systems are perceived as ‘green’ technologies and efficient solutions that industry stakeholders may find advantageous. However, there is inadequate relevant data on optimum extraction conditions and the effect of microwaves and ultrasound on cannabinoid and terpene yield. The effects of several independent variables, including samples-to-solvent ratio (s:s), extraction temperatures, extraction times, and duty cycles, on crude oil yield and concentration of cannabinoids and terpenes were examined. Response surface methodology (RSM) was used to optimize conditions as the established models evaluated and compared the effects of the dependent variables using quantitative results.

## 2. Results

### 2.1. Preliminary Ultrasound-Assisted Extraction and Microwave-Assisted Extraction Data

**Figure 1:**
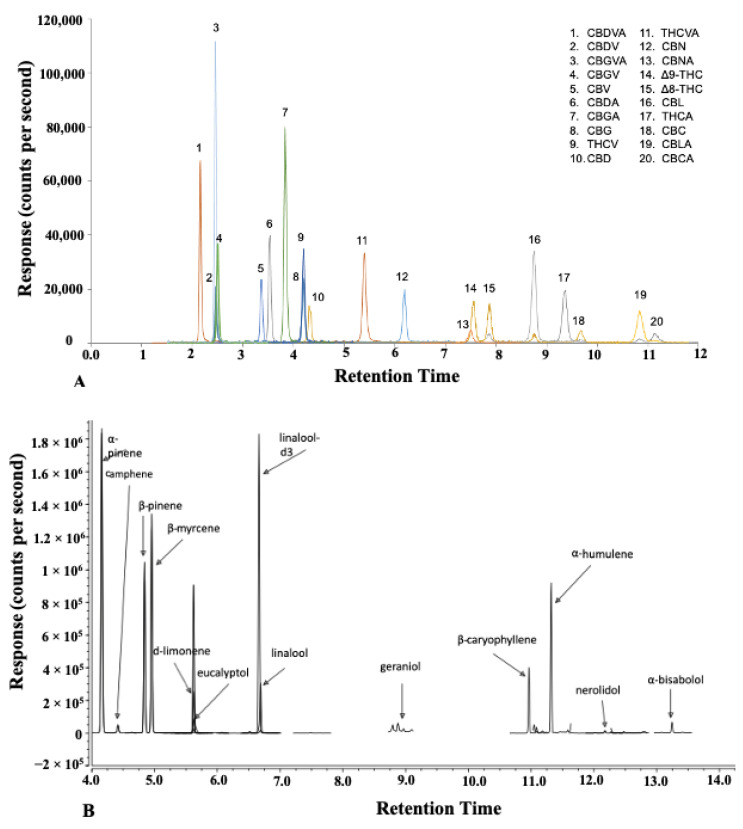
(**A**) LC-MS/MS chromatogram of cannabinoids and (**B**) GC-MS/MS chromatogram of terpenes.

Microwave- and ultrasound-assisted extraction methods were studied and compared. The selection of independent variables and their ranges for the extraction systems were based on preliminary experiments and a literature review of the probable effects of microwaves and ultrasound on the yield of cannabis oil, cannabinoids, and terpenes. The influence of the independent variables on the extraction of cannabis oil and the secondary metabolite profile by UAE and MAE was studied using the central composite rotatability design (CCRD). The central composite rotatable design was used because it consisted of five levels for each independent variable and was able to test fourth-order quadratic models. Major cannabinoid concentrations of the ground cannabis biomass are listed in Table 1. Cannabinoid and terpene concentrations were measured using the liquid chromatography-tandem mass spectrometer (LC-MS/MS) and gas chromatography-tandem mass spectrometer (GC-MS/MS), respectively. The total chromatographic run time was 18 min for the cannabinoids and 25 min for the terpenes (Figure 1). Cryo-ground biomass used for the study contained 17.9 g 100 g dry matter^−1^ (THCA), 0.17 g 100 g dry matter^−1^ (THC), 0.04 g 100 g dry matter^−1^ (CBDA), and 1.01 g 100 g dry matter^−1^ (THCVA).

The results of 31 and 20 experimental runs for UAE and MAE, respectively, carried out under the CCRD matrix for cannabis oil yields, cannabinoid concentration, and terpene concentration are presented in Table 2 and Table 3. Extraction conditions with 0 yield indicate that no extraction procedure was performed either due to a high concentration of sample or 0 extraction time. The reproducibility of the extraction data was verified through results obtained by the replication of the central points. No significant differences were observed in the responses of the central points for both extraction methods. 

Seven major cannabinoids, namely tetrahydrocannabinol (THC), tetrahydrocannabinolic acid (THCA), tetrahydrocannabivarin (THCVA), cannabigerol (CBG), cannabigerolic acid (CBGA), and cannabichromene acid (CBCA), were observed in all extracted samples (Table 2 and Table 3). Cannabidiol (CBD) and total CBD were not presented, as the concentration of CBD was below the limit of detection of the instrument and methodology. The findings demonstrate that the extracted cannabis oil yield ranged from 21.8 to 30.6 g 100 g dry matter^−1^ and 16.6 to 24.6 g 100 g dry matter^−1^ for UAE and MAE, respectively. Preliminary experiments showed that UAE extracted 16.6% more oil compared with MAE for samples extracted at 60 °C with a s: s of 1 g of cannabis biomass-to-15 mL of ethanol for 30 min. This significant effect (*p* < 0.05) in cannabis oil can be attributed to the structural damages and the improved solvent penetration into the plant matrix caused by the acoustic vibrations in UAE. Similar observations were made for the THCA (10.5%) and total terpenes (10.7%).

### 2.2. Effect of Ultrasound-Assisted and Microwave-Assisted Extraction Parameters on Cannabis Oil Yield

Optimizing the extraction yield is critical to the development of medicinal cannabis products, as increasing extract yield can reduce the overall production cost. The effects of four and three independent variables for UAE and MAE on the cannabis extraction yield were evaluated according to the significant coefficient (*p* < 0.05) of the full quadratic polynomial equation. The cannabis extraction yield for UAE was significantly (*p* < 0.05) influenced by sample (g) solvent (g)^−1^ and extraction temperature with first-order linear and second-order quadratic effects (extraction time^2^). A linear effect of sample (g) to solvent (g)^−1^ and a quadratic effect (s: s^2^) was observed for the extraction yield with MAE. According to these data, extending the UAE time from 10 min to 30 min resulted in a higher extraction yield (3.3%). 

Positive coefficient values (Table 4 and Table 5) for sample (g) to solvent (g)^−1^ for UAE and MAE showed that increasing the s: s significantly (*p* < 0.05) increases the extraction yield. A similar observation was made for the UAE extraction time. Thus, at a constant temperature of 40 °C, increasing the sample (g) solvent (g)^−1^ from 1:5 to 1:15 increases the yield by 15.6% and 33.8% for UAE and MAE, respectively, when samples were extracted for 30 min. Various extraction studies report that increasing sample (g) solvent (g)^−1^ can facilitate the mass transfer of compounds from the plant matrix into the solvent [30,31,32]. Sulaiman et al. (2017) showed that increasing the ratio of lindau (*Clinacanthus nutans*) leaves to ethanol from 70/30 (% *v*/*v*) to 90/10 (% *v*/*v*) increased the extraction yield by 20.8%. 

**Figure 2:**
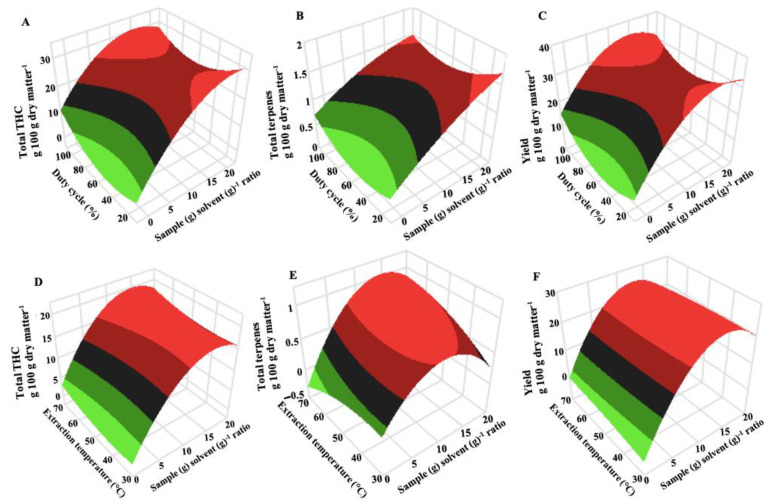
3D response surface plots illustrating the combined effects of sample (g) solvent (g)^−1^ and duty cycle (%) for ultrasound-assisted extraction (**A**–**C**) and sample (g) solvent (g)^−1^ and extraction temperature (°C) for microwave-assisted extraction (**D**–**F**) on the concentration (g 100 g dry matter^−1^) of total THC (**A**,**D**), total terpenes (**B**,**E**), and extraction yield (**C**,**F**).

The relationships between the independent parameters and extraction yield are illustrated in three-dimensional (3D) response surface plots (Figure 2). The extraction temperature insignificantly (*p* < 0.05) influenced the extraction yield for both UAE and MAE. An increase in the extraction temperature would confer either a negative or positive effect on extraction yield. This is evident with the slow linear increase in the extract yield from 28 to 28.6 g 100 g dry matter^−1^ for UAE and a slight decrease from 24.7 to 24.4 g 100 g dry matter^−1^ for MAE as the temperature rose from 40 to 60 °C using a sample (g)-to-solvent (g) ratio of 1:15. Increasing extraction temperature reduces solvent density, promoting an increase in the mass transfer rate and solute solubility, which improves the extraction yield. Irakli et al., (2018) showed that total phenolic compounds increased as the ultrasound extraction temperature increased from 25 to 60 °C for olives (*Olea europaea*). However, excessive extraction temperature degrades certain phytochemical compounds such as antioxidants (tannins, oxalate, etc.) and should be avoided [33,34]. 

### 2.3. Effect of Ultrasound-Assisted and Microwave-Assisted Extraction Parameters on Cannabinoids

Cannabinoids are mainly responsible for the therapeutic effects of cannabis [35]. Sample (g)-to-solvent (g) ratio had a linear significant (*p* < 0.05) effect on the major cannabinoids analyzed for both extraction systems, except for THC content after MAE (Table 4 and Table 5). This is supported by the low correlation (0.41) between MAE extraction yield and THC concentration and the parabolic shape of the curve (Figure 2). The THC content in MAE extracts was influenced by extraction temperature. For UAE, the quadratic effect of extraction time affected all cannabinoids; however, the quadratic effect of sample (g)-to-solvent (g) ratio only influenced THCA and CBG content. A positive coefficient value for sample (g)-to-solvent (g) ratio showed that increasing the sample (g)-to-solvent (g) ratio significantly (*p* < 0.05) increased cannabinoid concentration in extracts. Increasing the ratio from 1:5 to 1:15 increased the total THC in extracts by 37.7% and 19.3% for UAE and MAE, respectively, when samples were extracted for 10 min at 40 °C. This is likely due to cavitation bubbles with UAE and volumetric heating properties with MAE. 

Ultrasound-assisted extraction involves mechanical oscillating sound waves ranging from 20 kHz to 2 MHz that produce acoustic cavitation [12]. Acoustic cavitation is affected by s: s and physical properties of the solvent, such as viscosity, saturation vapor pressure, and surface tension [26,36]. Decreasing the sample (g)-to-solvent (g) ratio and viscosity of the solvent intensifies molecular interactions and thus hinders cavitation. The mechanical effect caused by the cavitation increases the permeability of the plant’s cell walls and improves the yield of cannabinoids [12,37,38]. Zakaria et al., 2021 showed that increasing the ratio of havil (*Mitragyna speciosa*) leaves to methanol by 66% increased the extraction yield and total phenolic content by 36.1% and 6.7%, respectively.

Microwave-assisted extraction uses microwaves to create heat and mass gradients [14,20]. Microwaves increase the kinetic energy of the solvent and improve the rate of penetration of the solvent into the solid matrix. Cannabinoids dissolve in the solvent and the solution diffuses to the surface of the solid. By natural or forced convection, the solution is transferred from the surface of the solid to the bulk medium. Increasing the sample-to-solvent ratio increases the amount of solvent diffusing into the solid matrix and hence, improves the concentration of secondary metabolites in the extracts [20,21].

### 2.4. Effect of Ultrasound-Assisted and Microwave-Assisted Extraction Parameters on the Total Terpenes

Terpenes are mainly responsible for the aroma of cannabis plants [39]. The greater terpene content of 23.8 to 25.8% observed with UAE extracts compared with MAE extracts can be attributed to the simultaneous action of the sonication that promoted the hydration and fragmentation reaction while expediting the rate of mass transfer of solutes to the extraction solvent and avoiding substantial solvent degradation. The sample-to-solvent ratio had a linear effect on total terpene content for both UAE and MAE. However, a quadratic effect (sample-to-solvent^2^) was only observed for MAE (Table 4 and Table 5). The main terpenes identified in the cannabis-extracted oil in this investigation were pinene, myrcene, eucalyptol, limonene, linalool, caryophyllene, and humulene. The observed terpenes are reported to have peppery, citrus, and hoppy mixed aroma [40,41]. As indicated in Figure 2, the duty cycle did not have a significant (*p* < 0.05) effect on terpene content. Increasing the duty cycle from 40 to 80% at a constant sample (g)-to-solvent (g) ratio of 1:5 and extraction temperature of 40 °C, caused a non-significant (*p* > 0.05) increase from 0.98 to 0.99 g 100 g dry matter^−1^. Extraction time and extraction temperature had similar minimal effects on the terpenes extracted. Terpenes have a low molecular mass and boiling point compared with other plant secondary metabolites [42]. They undergo thermal degradation with prolonged extraction time and increased temperature. Response surface plots (Figure 2) show that increasing the sample-to-solvent ratio from 1/5 to 1/15 caused a significant increase in terpenes by 11.2% and 23.8% for MAE and UAE, respectively, at a constant temperature (60 °C) and time (10 min). 

### 2.5. Model Fitting for Ultrasound-Assisted and Microwave-Assisted Extraction Systems

Responses consisting of THC, THCA, total THC, CBG, CBGA, total CBGA, THCVA, CBCA, total terpene content, and extraction yield for cannabis extracts for UAE and MAE were optimized using CCRD. Four and three second-order polynomial regression models were used to fit the experimental data for UAE and MAE, respectively. The models were included in the study to help explain the correlations between the independent variables and dependent variables and assist scale-up purposes. Regression coefficients for the intercept, linear, quadratic and interaction terms of the models were statistically analyzed and are presented in Table 4, Table 5 and Tables S1–S5 (Supplementary Materials). Based on ANOVA (Table 6) and the lack-of-fit data (0.05 to 0.98), models B and F are the best models for explaining the experimental data acquired for UAE and MAE, respectively. F ratios ranging from 1.5 to 5.8 and 3 to 30.5 for UAE and MAE, respectively, imply the significance of all the models. Notably, model E was not significant (*p* < 0.05) and does not explain the THCA and extract yield data. This is evident by the strong correlation (0.97) between THCA and the extraction yield. 

Revising model E to include only the sample-to-solvent (g) ratio and extraction temperature (°C) as independent parameters, was the ideal model for THCA and the yield. R^2^ values above 0.5 demonstrated a significant correlation between the CCRD design and the developed models. Apart from R^2^ values, the lack-of-fit analysis determines the validity of the models in which a *p*-value > 0.05 indicates that the model fits accurately with the experimental data. Since the lack-of-fit was only significant (*p* < 0.05) for the MAE extraction yield, this means that the quadratic polynomial model F does not accurately predict extract yield for cannabis oil using MAE. Further studies exploring other factors, such as microwave power, could be conducted and included in the model to expand our understanding of this method. The low coefficient of determination (R^2^) values for both models B and F showed that the models can be improved by considering the effects of other independent variables, such as ultrasound and microwave power densities on the extraction of cannabis oil, cannabinoids, and terpenes.

### 2.6. Optimal Experimental Conditions for Ultrasound-Assisted and Microwave-Assisted Extraction Systems for Cannabis

Ultrasound- and microwave-assisted extraction for cannabis were successfully optimized with a response surface methodology when evaluating the effects of the independent parameters of this study. All independent parameters were kept within the range for both extraction systems. Optimization was based on the maximum desirability function for the maximum yield of cannabinoids, total terpenes, and extracted cannabis oil. The desirability function consolidates all the responses into one response with a numerical value varying from 0 (one or more product characteristics are unacceptable) to 1 (all product characteristics are on target). The optimal independent experimental conditions for UAE and MAE at various conditions and the predicted responses at 95% confidence interval are presented in Table 7. UAE and MAE extractions of cannabis using a sample-to-solvent of 1:15 and 1:14.4, respectively, for 30 min at 60 °C were presented as the optimal conditions for maximum responses. Statistical analyses of the predicted responses showed significant (*p* < 0.05) differences between the extraction yields and secondary metabolite profiles for UAE and MAE. Under the optimal conditions, UAE extracts resulted in 14.4% more oil from cannabis biomass compared with MAE (Table 7). The concentration of total terpenes extracted was reduced by 14.7% when MAE was used. Compared with the reference ground sample (Table 1), the THCA concentration increased from 17.9 (g 100 g dry matter^−1^) to 28.5% and 20% with extraction efficiencies of 159.2% and 111.4% for ultrasound-assisted and microwave-assisted extraction, respectively. Extraction efficiency greater than 100% can be explained by the biosynthesis or conversion of other cannabinoids to THCA during the extraction process or variance due to the analytical method used.

### 2.7. Verification of Models for Ultrasound-Assisted and Microwave-Assisted Extraction Systems for Cannabis

Generated models for UAE and MAE for cannabis were verified by performing cannabis extraction using the optimal conditions (Table 7). The corresponding experimental values for the cannabinoid content, total terpenes, and extraction yields were determined and compared with the predicted results. The results showed a strong correlation ranging from 0.81 to 0.89 between the predicted and experimental values, which indicates the suitability of the models in predicting cannabinoid and terpenes profiles and extract yield for cannabis produced by the optimum UAE and MAE conditions.

### 2.8. Principal Component Analysis for Ultrasound-Assisted and Microwave-Assisted Extraction Systems for Cannabis

**Figure 3:**
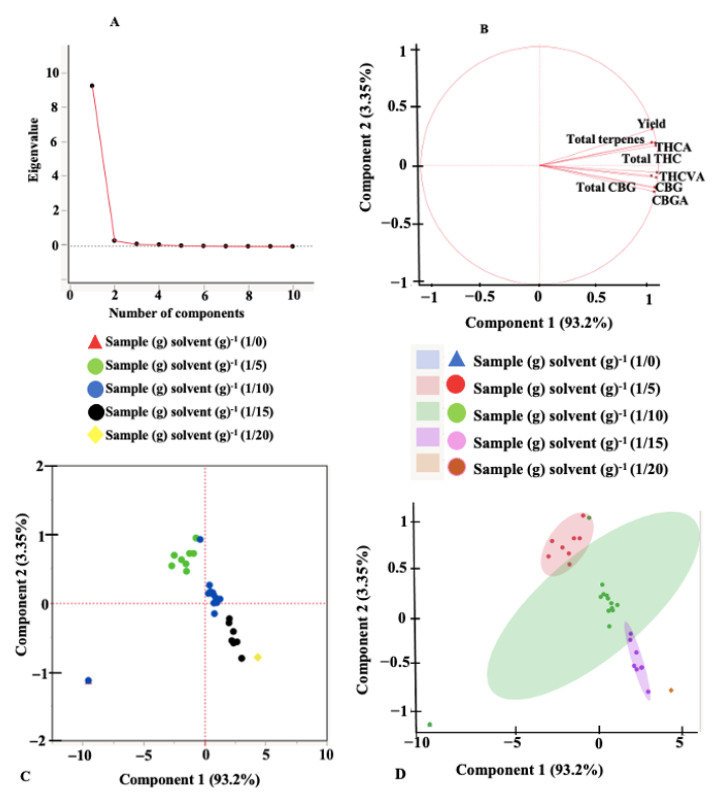
Principal component analysis plots. (**A**) scree plot, (**B**) loadings plot, (**C**) scores plot, and (**D**) scatterplot, for ultrasound-assisted extraction.

**Figure 4:**
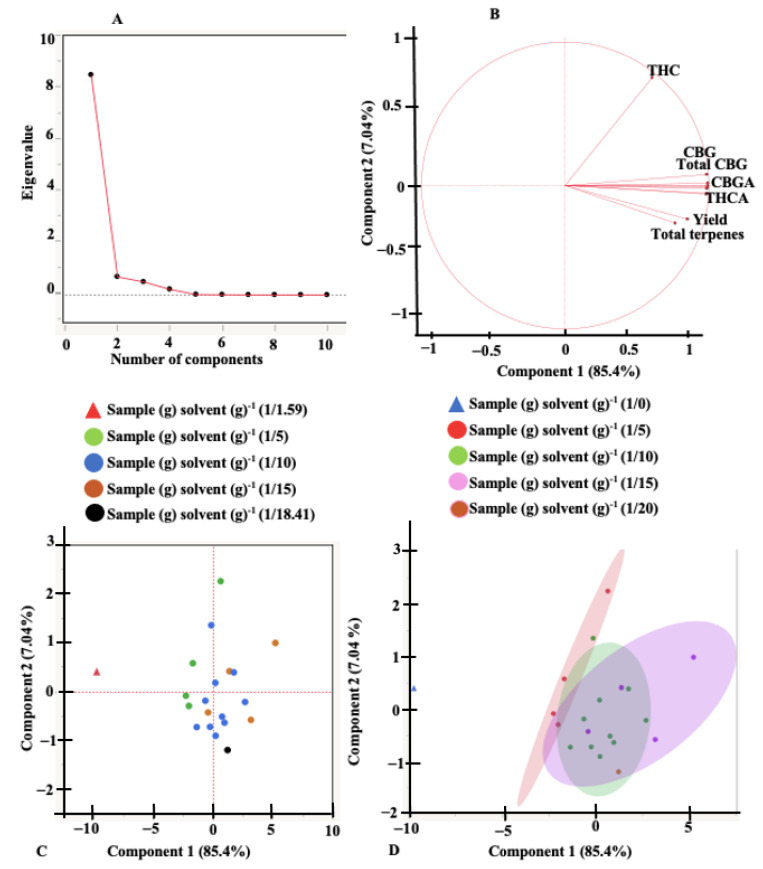
Principal component analysis plots. (**A**) scree plot, (**B**) loadings plot, (**C**) scores plot, and (**D**) scatterplot, for microwave-assisted extraction.

An exploratory principal component analysis (PCA) was performed to help identify correlation and dependencies between the independent variables and understand their effects on the responses. The scree plots, loading plots, score plots, and scatterplots for the different extraction systems are presented in Figure 3 and Figure 4. Scree plots are line plots of eigenvalues of principal components and are used to determine the number of principal components that are responsible for variations in the data during PCA [43]. Scree plots indicate that the first two principal components (PC) account for 96.55% of the total variance (PC1 = 93.2% and PC2 = 3.35%) for UAE and 92.44% of the total variance (PC1 = 85.4% and PC2 = 7.04%) for MAE. The loading plots provide information on how the various responses contribute to the variations accounted for by the principal components. Axes on the loading plot (1 to −1) describe how strongly the response influences the principal component. A positive value on the loading plot indicates a positive correlation between the response and the PC. Total THC, THCA, total terpenes, and the extraction yield directly influenced the variation observed by PC2 for UAE (Figure 3B) and inversely affected the variation accounted by PC2 for MAE. All dependent variables/responses identified in the extracts are important contributors to PC1 for both UAE and MAE, except the THC concentration under MAE. According to the loading plots, parameters positioned close to each other indicate a high positive correlation. Figure 3 and Figure 4 showed a strong correlation between all the dependent variables for both extraction systems except the THC concentration under MAE. The score and the scatter plots did not show any variation in the sample-to-solvent ratio for MAE. For UAE, however, there was a significant (*p* < 0.05) variation caused by the sample-to-solvent ratios.

## 3. Materials and Methods

### 3.1. Sample Preparation

Harvested inflorescence from three cannabis accessions, Qrazy Train, Qrazy Apple, and Qrazy Angel, that were cultivated indoors under the same growing conditions were obtained from EXKA Inc. (Mirabel, QC, Canada). Inflorescences were pre-frozen at −20 °C for 24 h before transferring to a laboratory-scale vacuum freeze-dryer (Martin Christ Gefriertrocknungsanlagen GmbH Gamma 1–16 LSCplus, Osterode, Lower Saxony, Germany) with a condenser temperature of −55 °C. Freeze-drying was carried out at 10 °C for 24 h at 0.85 mbar. The initial moisture content of the inflorescence ranged from 78.52 to 80.48% (wb). Using a previously described method for hops [44], the freeze-dried inflorescences of the different accessions were mixed and cryo-ground to uniform particle size (0.25–0.5 mm) using liquid nitrogen and a mortar and pestle. Ground samples were kept in clean plastic bags, homogenized by hand mixing and shaking, and stored at either −20 °C or −40 °C before extraction and analysis.

### 3.2. Reagents

Food-grade ethanol was purchased from Commercial Alcohols (Brampton, Ontario, Canada). Reference standards of cannabinoids and isotopically labeled cannabinoids were purchased from Cerilliant (Round Rock, TX, USA). All neutral cannabinoids including Δ9-THC (tetrahydrocannabinol), Δ8-THC, CBD (cannabidiol), CBG (cannabigerol), CBN (cannabinol), CBC (cannabichromene), THCV (tetrahydrocannabivarin), CBDV (cannabidivarin), CBGV (cannabigerivarin), and CBV (cannabivarin) were provided at 1.0 mg mL^−1^ in methanol. CBL (cannabicyclol) was provided at 1.0 mg mL^−1^ in acetonitrile. The acidic cannabinoids, including Δ9-THCA (tetrahydrocannabinolic acid), CBDA (cannabidiolic acid), CBGA (cannabigerolic acid), CBNA (cannabinolic acid), CBCA (cannabichromenic acid), THCVA (tetrahydrocannabivarin acid), CBDVA (cannabidivarinic acid), and CBGVA (cannabigerovarinic acid), were provided at 1.0 mg mL^−1^ in acetonitrile. CBLA (cannabicyclolic acid) was provided at 0.5 mg mL^−1^ in acetonitrile.

Isotopically labeled cannabinoids, including Δ9-THC-d_3_, CBD-d_3_, CBN-d_3_, and CBG-d_3_, were provided at 0.1 mg mL^−1^ in methanol while Δ9-THCA-d_3_, CBGA-d_3_, and CBCA-d_3_ were provided at 0.1 mg mL^−1^ in acetonitrile. THC-d_3_ was used as the internal standard for Δ9-THC, Δ8-THC, THCV, CBC, and CBL. THCA-d_3_ was used for THCA, CBNA, and THCVA. CBD-d_3_ was used for CBD, CBDA, CBDV, and CBDVA. CBN-d_3_ was used for CBN and CBV. CBG-d_3_ was used for CBG and CBGV. CBGA-d_3_ was used for CBGA and CBGVA and CBCA-d_3_ was used for CBCA and CBLA. Ultrapure water was collected from a Millipore Milli-Q Advantage A10 mixed bed ion exchange system fed with reverse osmosis domestic water (Jaffrey, New Hampshire, US). Optima^®^ grade acetonitrile, methanol, and formic acid were procured from Fisher Scientific (Fair Lawn, NJ, USA).

Terpene reference standards were purchased from Restek (Bellefonte, PA, US) and provided at 2.5 mg mL^−1^ in isopropanol. Isotopically labeled terpene (±)-linalool-d3 (vinyl-d3) was purchased from CDN Isotopes (Pointe-Claire, QC, Canada) and used as an internal standard. Hexane (HPLC Plus, ≥95%) was purchased from Millipore-Sigma (Oakville, ON, Canada).

### 3.3. Extraction Procedures

Ultrasound-assisted (UAE) and microwave-assisted (MAE) extractions were carried out with different sample (g)-to-solvent (g) ratios, extraction temperatures, and extraction times. The influence of the duty cycle of the ultrasound was used as an independent variable for the ultrasound-assisted extraction of cannabis oil. Sample (g)-to-solvent (g) ratios used for this study were calculated by varying the mass of cannabis biomass into 40 mL ethanol with Equation (1).

(1) $$\mathrm{Mass}\;\mathrm{of}\;\mathrm{cannabis}\;\mathrm{biomass}=40\;\mathrm{mL}\times \frac{\mathrm{density}\;\mathrm{of}\;\mathrm{ethanol}\;\left(0.789\frac{g}{\mathrm{mL}}\right)}{\mathrm{mass}\;\mathrm{of}\;\mathrm{ethanol}\;\left(g\right)}\;$$

#### 3.3.1. Ultrasound-Assisted Extraction

**Figure 5:**
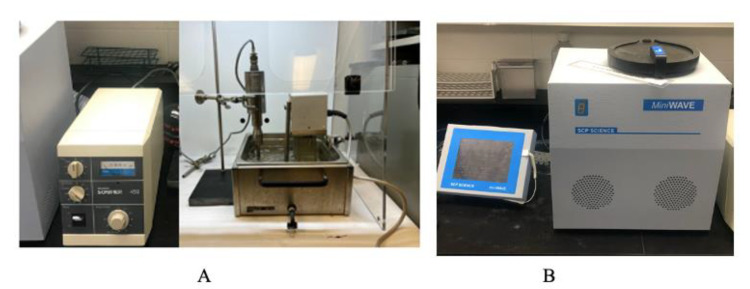
Branson Sonifier 450 ultrasound (**A**) and mini-wave microwave (**B**) extraction systems used for the study.

A Branson Sonifier 450 ultrasound system (Marshall Scientific, Hampton, VI, USA) with a fixed working frequency of 20 kHz and an electric power output of 450 W was used for the UAE of crude cannabis oil (Figure 5A). The ultrasound system consisted of an ultrasound generator, a transducer, and an ultrasound probe. Cannabis biomass mixed with ethanol was placed in a 50-mL beaker positioned in a water bath with a heating coil system to maintain the extraction temperature. The ultrasonic emitter was immersed 1 cm into the solution, as previously described, [45] and turned on. Acoustic cavitation (creation, growth, and implosion of gas bubbles under the ultrasonic treatment) was observed, and the duty cycle was set at the desired level, ranging from 20 to 100%. The duty cycle is the percentage of the total ultrasound extraction time during which the ultrasound signal and power are “on”.

#### 3.3.2. Microwave-Assisted Extraction

Microwave-assisted extraction of crude cannabis oil was performed in a multi-mode (closed) mini-wave microwave unit (SCP Science, Baie-D’Urfe, QC, Canada). The system consists of a touchscreen controller that is USB-connected with the microwave module (digestion chamber) (Figure 5B). The magnetron is located at the base of the module to ensure even distribution of the microwave energy across the digestion chamber. The module has six equidistant and radially constructed 75-mL vessels in a non-rotating digestion rack. Quartz vessels were used for the microwave extraction process. The average real-time operating temperature was monitored using six infrared sensors located on the side walls of the oven. Irradiation frequency and power were 2.45 GHz and 1000 W, respectively. The duration of irradiation included ramp time (time to reach the target process temperature, set at 5 min for all experiments) and hold time (elapsed time while irradiating the sample at a set temperature). The unit had a forced air ventilation system for cooling. 

### 3.4. Calculation of Extraction Yield and Efficiency

After extraction processes, each extract containing the solvent and cannabis biomass mixture was subjected to vacuum filtration using Whatman 4 filter paper (Sigma Aldrich, St. Louis, MO, USA). The mass of the crude cannabis oil was derived using a vacuum rotary evaporator, operating at 35 rpm and 50 °C, to evaporate ethanol present in the extract. The extraction yield of the crude cannabis oil was calculated using Equation (2). However, the extraction efficiency was calculated based on the concentration of the major cannabinoid (THCA) using Equation (3).

(2) $$\mathrm{Yield}\;\left(g\;100{\;g\;\mathrm{dry}\;\mathrm{matter}}^{-1}\right)=\frac{\mathrm{mass}\;\mathrm{of}\;\mathrm{crude}\;\mathrm{cannabis}\;\mathrm{oil}\;\left(g\right)}{\mathrm{mass}\;\mathrm{of}\;\mathrm{dried}\;\mathrm{sample}\;\left(100\;g\right)}\;$$

(3) $$\mathrm{Efficiency}\;\left(\%\right)=\frac{\mathrm{Concentration}\;\mathrm{of}\;\mathrm{THCA}\;\mathrm{in}\;\mathrm{extract}\;\left(\frac{g}{100\;g\;\mathrm{dry}\;\mathrm{matter}}\right)}{\mathrm{Initial}\;\mathrm{concentration}\;\mathrm{of}\;\mathrm{THCA}\;\mathrm{in}\;\mathrm{cryo}-\mathrm{ground}\;\mathrm{sample}\;\left(\frac{g}{100\;g\;\mathrm{dry}\;\mathrm{matter}}\right)}\times 100\%\;$$

### 3.5. Cannabinoid Analyses by LC-MS/MS

Cannabinoid analysis method developed and described previously by the National Research Council of Canada was modified and used for this study [46,47]. Extracted crude cannabis oil samples were centrifuged at 5000 rpm for 5 min. An aliquot of the supernatant was diluted in methanol based on the initial sample biomass (Table 8) used for the extraction (this sample is referred to as the diluted cannabis extract). Samples, standards, and quality control (QC) samples (100 μL) were transferred to high-pressure liquid chromatography (HPLC) vials containing glass inserts. The internal standard (50 μL, 500 ng mL^−1^ in methanol) was added prior to injection onto the liquid chromatography tandem mass spectrometer (LC-MS/MS) system. The LC-MS/MS system consisted of an HPLC (Ultimate3000; Thermo Fisher Scientific, Waltham, MA, USA) coupled to a triple quadrupole mass spectrometer (TSQ Quantiva; Thermo Fisher Scientific, Waltham, MA, USA). Chromatographic separation was carried out on a C18 bonded phase column (Accucore C18, 150 mm × 2.1 mm i.d. with 2.6 μm particle size; Thermo Fisher Scientific, Waltham, MA, USA) maintained at 40 °C, and the mobile phases consisted of water/formic acid and acetonitrile/formic acid both mixed in a 1000:1 volume ratio.

The MS/MS detection of cannabinoids was performed via electrospray ionization in positive ion mode using quasi-molecular ion-to-product ion transitions. External calibration standard solutions containing 20 cannabinoids were prepared in methanol at concentrations of 10, 20, 100, 1000, 6000, 9000 and 10,000 ng mL^−1^) with quality control samples prepared at 30, 1500 and 8000 ng mL^−1^. Linear regression, weighted 1/x^2^, was used for calibration, with the peak area ratio of cannabinoid and internal standard as the response variable. 

### 3.6. Terpene Analysis

For terpene analysis, extracted crude cannabis oil samples were centrifuged at 5000 rpm for 5 min. An aliquot of the supernatant was diluted in hexane based on the initial sample biomass (Table 8) used for the extraction (referred to as the diluted cannabis extract). Samples, standards, and QC samples (150 μL) were transferred to HPLC vials containing glass inserts and the internal standard (50 μL, 1 μg mL^−1^ of linalool-d_3_ in hexane) was added before injection onto the gas chromatography-tandem mass spectrometer (GC-MS/MS) system (Trace 1310 GC coupled to a TSQ 9000 Triple Quadrupole MS/MS; Thermo Fisher Scientific, Waltham, MA, USA).

Chromatographic separation of the analytes was obtained using the TraceGOLD TG-5SilMS column (30 m × 0.25 mm i.d. with 0.25 μm film thickness; Thermo Fisher Scientific, Waltham, MA, USA) and helium as the carrier gas. The SSL inlet temperature was held at 250 °C with a deactivated splitless quartz wool single taper liner (78.5 mm × 4 mm i.d. × 6.3 mm o.d.; Thermo Fisher Scientific, Waltham, MA, USA). A constant inlet flow of 1.5 mL min^−1^ with a split flow of 15 mL min^−1^ and a split ratio of 10 was used. Selected reaction monitoring (SRM) scan type with electron impact ionization mode was used for the tandem mass spectrometer, while the ion source temperature and MS transfer line temperature were held at 300 °C and 250 °C, respectively. The temperature program for the GC oven can be found in Table 9.

Calibration curves (0.005–2.5 µg mL^−1^) were generated using weighted linear regression (1/x) of the peak area ratios (analyte/internal standard) versus the concentration of the calibration standards. The concentration of individual terpenes in extracts was determined using the appropriate calibration curve for the metabolite using the resulting peak area ratios.

### 3.7. Experimental Design

A five-level-by-four-variables and five-level-by-three-variables central composite rotatable statistical design (CCRD) with uniform precision was used for ultrasound-assisted extraction and microwave-assisted extraction, respectively. CCRD was used to assess and compare the effects of the different extraction conditions on the total yield of cannabis crude oil, cannabinoids, and terpenes. It comprised 16 combinations of factorial values, 8 combinations of axial values, and 7 combinations of central values for ultrasound-assisted extraction (Table 10). For MAE, combinations for the factorial, axial, and central values were 8, 6, and 6, respectively (Table 10); this was due to the reduced number of variables for this extraction method. Axial points were fixed at a distance (α = 2^k/4^, where k represents the number of variables) from the center to ensure rotatability. Axial combinations allow for the inclusion of quadratic terms in the response surface model. Replication of a central point ensures a greater uniformity in the precision of response estimation over the experimental design.

### 3.8. Statistical Analysis

The analysis of the independent variables’ effect was assessed using JMP software (JMP 4.3 SAS Institute Inc.). The least-square multiple regression method was used to evaluate the relationship between the independent and dependent variables. Four and three multiple regression equations were used to fit the second-order polynomial model based on the experimental data for ultrasound-assisted extraction and microwave-assisted extraction, respectively (Table 11). Models A and E represent the full regression model for UAE and MAE, respectively. It includes all the independent terms, their interactions, and quadratic terms. The reduced regression models for UAE (models B, C, and D) and MAE (models F and G) were evaluated by controlling one independent parameter. The analysis of variance (ANOVA) was used to investigate the statistical significance of the regression coefficients by conducting the Fisher’s F-test at a 95% confidence level. The statistical significance of the model was improved through a “backward elimination” process, deleting non-significant dependent terms (*p* > 0.05). A response surface plot was obtained using the fitted model. Optimal conditions for MAE and UAE for the dependent variables were determined based on modelling and desirability function and principal component analysis (PCA) using JMP software (JMP 4.3 SAS Institute Inc., Cary, NC, USA).

### 3.9. Model Verification

To verify the model, three experiments were conducted using optimal extraction conditions with the highest desirability. The experimental and predicted values were compared with determine the validity of the model.

## 4. Conclusions

The depenalization of the cannabis industry in Canada has intensified cannabis production and driven sales of cannabis and cannabis products for medical and recreational adult use. Although some commercial entities have developed efficient extraction systems to improve the safety and potency of cannabis, most of these novel systems have not been optimized for maximum extraction yield and concentration of secondary metabolites. This study optimized for maximum extraction efficiency, using CCRD as a function of several independent variables, namely samples-to-solvent ratio, extraction temperatures, extraction times, and duty cycles. Cannabis samples were extracted using ultrasound-assisted and microwave-assisted extraction. UAE and MAE extraction of cannabis using a sample-to-solvent ratio of 1:15 and 1:14.4, respectively, for 30 min at 60 °C were presented as the optimal conditions for maximum responses with maximum desirability of 0.83% and 0.75% for UAE and MAE, respectively. UAE increased the crude oil yield, cannabinoid concentration, and total terpene extracted by 14.39%, 13.21–39.24%, and 14.67% respectively, compared with MAE. Developed predictive models for all responses yielded predictable and reproducible results, and the verification of the models showed a close agreement between the experimental values and the predicted values, with a strong correlation ranging from 0.81 to 0.89. Scree plots under PCA indicated that the first two principal components account for 96.55% of the total variance (PC1 = 93.2% and PC2 = 3.35%) for UAE and 92.44% of the total variance (PC1 = 85.4% and PC2 = 7.04%) for MAE. The data showed a significant (*p* < 0.05) variation caused by the sample-to-solvent ratios for only the UAE. Further research studies on ethanol recovery using centrifugation, mechanical press system, and vacuum filtration must be conducted to help reduce the operational cost for cannabis industries.

## Figures and Tables

**Table 1:** Major cannabinoid and terpene concentrations (g 100 g dry matter^−1^) present in cryo-ground sample.

Metabolite	Concentration (g 100 g Dry Matter^−1^)
Tetrahydrocannabinol (Δ9-THC)	0.17 ± 0.11
Tetrahydrocannabinolic acid (THCA)	17.92 ± 6.24
Cannabidiolic acid (CBDA)	0.04 ± 0.03
Cannabigerol (CBG)	0.09 ± 0.05
Cannabigerolic acid (CBGA)	0.27 ± 0.18
Cannabinolic acid (CBNA)	0.02 ± 0.01
Cannabichromenic acid (CBCA)	0.38 ± 0.25
Tetrahydrocannabivarin (THCV)	0.01 ± 0
Tetrahydrocannabivarin acid (THCVA)	1.01 ± 0.4
Total THC	15.87 ± 0.56
Total CBG	0.32 ± 0.02

**Table 2:** Crude cannabis oil yield and concentration of cannabinoids and total terpenes obtained from cryo-ground cannabis subjected to ultrasound-assisted extraction.

Independent Variables				Response/Dependent Variables (g 100 g Dry Matter^−1^)									
X_1_	X_2_	X_3_	X_4_	THC	THCA	Total THC	CBG	CBGA	Total CBGA	THCVA	CBCA	Total Terpenes	Yield
1/0	20	60	50	0	0	0	0	0	0	0	0	0	0
1/5	10	40	40	0.44	17.06	15.4	0.08	0.23	0.29	0.80	0.09	0.98	21.80
1/5	10	40	60	0.47	18.4	16.61	0.08	0.23	0.28	0.87	0.09	0.93	23.42
1/5	10	80	40	0.57	19.34	17.53	0.09	0.24	0.31	0.96	0.10	0.99	24.49
1/5	10	80	60	0.59	20.21	18.31	0.1	0.26	0.33	0.99	0.11	0.97	25.63
1/5	30	40	40	0.55	20.36	18.4	0.09	0.27	0.33	1.16	0.12	0.96	24.41
1/5	30	40	60	0.57	22.58	20.38	0.11	0.23	0.32	1.18	0.12	0.92	25.4
1/5	30	80	40	0.59	24.24	21.85	0.12	0.25	0.34	1.29	0.11	0.89	25.99
1/5	30	80	60	0.61	25.22	22.73	0.12	0.24	0.33	1.32	0.11	0.99	26.11
1/10	0	60	50	0	0	0	0	0	0	0	0	0	0
1/10	20	20	50	0.63	26.14	23.55	0.13	0.24	0.34	1.31	0.13	1.01	26.35
1/10	20	60	30	0.66	26.34	23.75	0.14	0.35	0.44	1.37	0.15	1	26.5
1/10	20	60	50	0.74	26.4	22.04	0.15	0.36	0.44	1.42	0.13	1.05	26.9
1/10	20	60	50	0.79	26.31	24.74	0.15	0.4	0.47	1.47	0.15	1.04	27.53
1/10	20	60	50	0.72	26.44	22.93	0.16	0.37	0.45	1.48	0.14	1.05	27.22
1/10	20	60	50	0.77	26.67	20.58	0.15	0.37	0.4	1.42	0.12	1.05	26.58
1/10	20	60	50	0.78	25.66	23.29	0.16	0.39	0.48	1.43	0.14	1.03	26.5
1/10	20	60	50	0.77	26.05	23.52	0.15	0.39	0.47	1.41	0.14	1.05	26.9
1/10	20	60	50	0.73	26.53	23	0.15	0.37	0.45	1.47	0.14	1.03	27.44
1/10	20	60	70	0.84	26.3	23.91	0.16	0.38	0.49	1.62	0.14	1.17	27.85
1/10	20	100	50	0.80	25.82	23.44	0.16	0.37	0.48	1.6	0.13	1.11	27.13
1/10	40	60	50	0.79	26.1	23.68	0.15	0.36	0.47	1.63	0.14	1.1	26.81
1/15	10	40	40	0.89	27.15	24.7	0.17	0.47	0.58	1.64	0.14	1.28	27.96
1/15	10	40	60	0.91	27.77	25.27	0.16	0.47	0.58	1.68	0.24	1.22	28.57
1/15	10	80	40	0.93	27.95	25.45	0.17	0.45	0.56	1.73	0.14	1.19	28.71
1/15	10	80	60	0.96	28.43	25.89	0.17	0.48	0.6	1.76	0.15	1.05	29.25
1/15	30	40	40	0.93	28.12	25.59	0.18	0.49	0.6	1.75	0.16	1.12	28.91
1/15	30	40	60	0.97	28.21	25.7	0.18	0.44	0.57	1.79	0.17	1.12	29.38
1/15	30	80	40	1.14	28.45	26.1	0.18	0.45	0.57	1.81	0.17	1.16	29.52
1/15	30	80	60	1.15	28.74	26.35	0.19	0.47	0.6	1.88	0.19	1.12	29.86
1/20	20	60	50	1.22	29.19	26.82	0.2	0.55	0.68	2.05	0.21	1.6	30.63

X_1_ (Sample (g) solvent (g)^−1^), X_2_ (Extraction time (min)), X_3_ (Duty cycle (%)), and X_4_ (Extraction temperature (°C)) are the independent variables.

**Table 3:** Crude cannabis oil yield and concentration of cannabinoids and total terpenes obtained from cryo-ground cannabis subjected to microwave-assisted extraction.

Independent Variables			Response/Dependent Variables (g 100 g Dry Matter^−1^)									
X_1_	X_2_	X_4_	THC	THCA	Total THC	CBG	CBGA	Total CBGA	THCVA	CBCA	Total Terpenes	Yield
1/1.59	20	50	0	0	0	0	0	0	0	0	0	0
1/5	10	40	0.39	13.17	11.94	0.06	0.19	0.23	0.70	0.24	0.73	16.64
1/5	10	60	1.32	16.12	15.45	0.09	0.24	0.30	0.87	0.33	0.79	19.18
1/5	30	40	0.45	11.95	10.93	0.06	0.18	0.21	0.67	0.25	0.73	16.35
1/5	30	60	0.63	13.42	12.40	0.07	0.20	0.24	0.73	0.24	0.69	14.72
1/10	3	50	0.44	15.29	13.85	0.08	0.22	0.27	0.83	0.30	0.92	23.97
1/10	20	33	0.38	16.68	15.01	0.08	0.25	0.30	0.91	0.31	0.97	22.01
1/10	20	50	0.56	17.16	15.60	0.09	0.25	0.30	0.93	0.32	1.02	24.13
1/10	20	50	0.61	14.48	13.31	0.07	0.20	0.25	0.80	0.29	0.90	23.42
1/10	20	50	0.74	19.56	17.90	0.10	0.28	0.35	1.10	0.39	0.67	24.05
1/10	20	50	0.46	12.81	11.70	0.06	0.19	0.23	0.71	0.25	0.98	23.81
1/10	20	50	0.71	21.03	19.16	0.10	0.30	0.37	1.20	0.42	1.07	23.66
1/10	20	50	0.53	18.05	16.36	0.08	0.26	0.31	1.00	0.35	0.97	24.61
1/10	20	67	1.10	14.16	13.52	0.08	0.21	0.27	0.79	0.28	0.83	24.05
1/10	37	50	0.78	15.04	13.96	0.08	0.23	0.28	0.87	0.30	0.96	25.00
1/15	10	40	0.35	16.48	14.80	0.08	0.23	0.28	0.90	0.32	0.37	25.36
1/15	10	60	0.83	18.18	16.78	0.09	0.27	0.33	1.03	0.35	0.89	23.70
1/15	30	40	0.56	22.42	20.22	0.11	0.32	0.39	1.27	0.45	0.96	24.76
1/15	30	60	1.11	25.26	23.27	0.13	0.39	0.47	1.43	0.50	1.00	24.40
1/18.41	20	50	0.39	18.00	16.18	0.09	0.26	0.32	1.04	0.35	1.12	24.56

X_1_ (Sample (g) solvent (g)^−1^), X_2_ (Extraction time (min)), and X_4_ (Extraction temperature (°C)) are the independent variables.

**Table 4:** Matrix of the central composite rotatable statistical design (CCRD) and observed responses (Y_j_) for ultrasound-assisted extraction using model A (Y_j_ = β_0_ + β_1_X_1_ + β_2_X_2_ + β_3_X_3_ + β_4_X_4_ + β_11_X_1_X_1_ + β_22_X_2_X_2_ + β_33_X_3_X_3_ + β_44_X_4_X_4_ + β_12_X_1_X_2_ + β_13_X_1_X_3_ + β_14_X_1_X_4_ + β_23_X_2_X_3_ + β_24_X_2_X_4_ + β_34_X_3_X_4_).

Response/Dependent Variables		Regression Model Effect Parameters														
		Intercept	Linear				Interaction						Quadratic			
		β_0_	β_1_	β_2_	β_3_	β_4_	β_12_	β_13_	β_23_	β_14_	β_24_	β_34_	β_11_	β_22_	β_33_	β_44_
THC	Coefficient	0.76	0.25	0.1	0.05	0.02	0.02	0.01	0.01	0.001	−0.001	−0.002	−0.01	−0.07	0.01	0.02
	*p* value	<0.0001 *	<0.0001 *	0.01 *	0.11	0.43	0.66	0.79	0.82	0.99	0.99	0.96	0.65	0.02 *	0.59	0.38
THCA	Coefficient	26.29	4.82	2.99	0.51	0.28	−0.95	−0.52	0.11	−0.25	0.02	−0.1	−2.17	−2.56	0.68	0.76
	*p* value	<0.0001 *	0.0001 *	0.01 *	0.59	0.77	0.42	0.66	0.92	0.83	0.99	0.93	0.02 *	0.01 *	0.44	0.39
Total THC	Coefficient	22.87	4.48	2.72	0.50	0.27	−0.82	−0.44	0.11	−0.22	0.01	−0.01	−1.68	−2.07	0.84	0.93
	*p* value	<0.0001 *	<0.0001 *	0.01 *	0.57	0.76	0.45	0.68	0.92	0.84	0.99	0.93	0.05	0.02 *	0.3	0.26
CBG	Coefficient	0.15	0.04	0.02	0.01	0.003	−0.002	−0.003	0.001	−0.002	0.002	0.001	−0.01	−0.02	0.001	0.003
	*p* value	<0.0001 *	<0.0001 *	0.01 *	0.17	0.52	0.73	0.57	0.91	0.73	0.73	0.91	0.02 *	0.01 *	0.76	0.54
CBGA	Coefficient	0.38	0.12	0.03	0.01	0.001	−0.003	−0.003	−0.003	0.002	−0.01	0.01	−0.01	−0.04	−0.01	0.01
	*p* value	<0.0001 *	<0.0001 *	0.05	0.45	0.93	0.86	0.86	0.86	0.92	0.66	0.61	0.32	0.01 *	0.65	0.52
Total CBG	Coefficient	0.45	0.15	0.04	0.02	0.01	−0.01	−0.01	−0.003	0.003	−0.004	0.01	−0.01	−0.04	0.004	0.02
	*p* value	<0.0001 *	<0.0001 *	0.02 *	0.4	0.76	0.8	0.8	0.89	0.89	0.84	0.71	0.42	0.03 *	0.80	0.29
THCVA	Coefficient	1.44	0.4	0.21	0.06	0.03	−0.06	−0.01	−0.002	0.002	−0.001	−0.001	−0.07	−0.12	0.04	0.05
	*p* value	<0.0001 *	<0.0001 *	0.01 *	0.23	0.48	0.35	0.81	0.98	0.98	0.99	0.99	0.14	0.01 *	0.39	0.28
CBCA	Coefficient	0.14	0.04	0.02	−0.002	0.01	−0.003	−0.004	0.004	0.01	−0.01	−0.004	−0.003	−0.01	0.002	0.01
	*p* value	<0.0001 *	<0.0001 *	0.03 *	0.75	0.41	0.69	0.58	0.58	0.31	0.48	0.58	0.53	0.05 *	0.68	0.3
Total terpenes	Coefficient	1.04	0.2	0.08	0.001	0.004	−0.01	−0.02	0.02	−0.01	0.02	0.003	−0.03	−0.09	0.03	0.04
	*p* value	<0.0001 *	0.0021 *	0.17	0.98	0.95	0.92	0.81	0.82	0.83	0.79	0.96	0.55	0.08	0.5	0.43
Yield	Coefficient	27.01	4.01	2.64	0.47	0.36	−0.21	−0.29	−0.18	−0.12	−0.12	−0.1	−1.89	−2.37	0.96	1.07
	*p* value	<0.0001 *	0.0045 *	0.04 *	0.70	0.77	0.89	0.85	0.9	0.94	0.93	0.95	0.11	0.05 *	0.4	0.35

Where Y_j_ represents the predicted response (dependent variables), the model intercept (β_0_), linear terms (β_1_, β_2_, β_3_, and β_4_), interaction terms (β_11_, β_22_, β_33_, and β_44_) and quadratic terms (β_12_, β_13_, β_14_, β_23_, β_24_, and β_34_), and X_1_ (Sample (g) solvent (g)^−1^), X_2_ (Extraction time (min)), X_3_ (Duty cycle (%)), and X_4_ (Extraction temperature (°C)) are the independent variables.* Independent effects are statistically significant if *p* < 0.05.

**Table 5:** Matrix of the central composite rotatable statistical design (CCRD) and observed responses (Y_j_) for microwave-assisted extraction using model E (Y_j_ = β_0_ + β_1_X_1_ + β_2_X_2_ + β_4_X_4_ + β_11_X_1_X_1_ + β_22_X_2_X_2_ + β_44_X_4_X_4_ + β_12_X_1_X_2_ + β_14_X_1_X_4_ + β_24_X_2_X_4_).

Response/Dependent Variables		Regression Model Effect Parameters									
		Intercept	Linear			Interaction			Quadratic		
		β_0_	β_1_	β_2_	β_3_	β_12_	β_13_	β_23_	β_11_	β_22_	β_33_
THC	Coefficient	0.6	0.05	0.03	0.25	0.14	−0.01	−0.09	−0.1	0.04	0.09
	*p* value	<0.0001 *	0.33	0.55	<0.0001 *	0.06	0.88	0.23	0.07	0.40	0.1
THCA	Coefficient	17.05	4.24	0.64	0.35	2.12	0.02	−0.04	−2.01	0.17	0.26
	*p* value	<0.0001 *	0.002 *	0.53	0.73	0.13	0.99	0.97	0.06	0.86	0.79
Total THC	Coefficient	15.55	3.78	0.59	0.55	2.00	0.01	−0.12	−1.87	0.19	0.32
	*p* value	<0.0001 *	0.002 *	0.53	0.55	0.12	0.93	0.92	0.06	0.83	0.73
CBG	Coefficient	0.08	0.02	0.003	0.01	0.01	−0.001	−0.001	−0.01	0.003	0.003
	*p* value	<0.0001 *	0.002 *	0.48	0.32	0.11	0.85	0.85	0.07	0.57	0.57
CBGA	Coefficient	0.24	0.06	0.01	0.01	0.03	0.01	0.002	−0.03	0.01	0.01
	*p* value	<0.0001 *	0.002 *	0.40	0.58	0.12	0.80	1	0.07	0.73	0.64
Total CBG	Coefficient	0.30	0.08	0.01	0.01	0.04	0.004	−0.001	−0.04	0.01	0.01
	*p* value	<0.0001 *	0.001 *	0.45	0.46	0.1	0.87	0.96	0.06	0.76	0.61
THCVA	Coefficient	0.95	0.25	0.05	0.02	0.12	0.01	−0.01	−0.11	0.01	0.01
	*p* value	<0.0001 *	0.001 *	0.39	0.68	0.13	0.92	0.89	0.07	0.86	0.86
CBCA	Coefficient	0.33	0.08	0.01	0.01	0.05	0.003	−0.01	−0.04	0.005	0.003
	*p* value	<0.0001 *	0.002 *	0.47	0.69	0.11	0.9	0.7	0.06	0.81	0.89
Total terpenes	Coefficient	0.94	0.16	0.05	0.03	0.1	0.07	−0.07	−0.14	−0.003	−0.02
	*p* value	<0.0001 *	0.02 *	0.42	0.68	0.22	0.40	0.37	0.03 *	0.96	0.77
Yield	Coefficient	23.92	5.32	−0.21	0.17	0.61	−0.37	−0.36	−3.92	0.39	−0.12
	*p* value	<0.0001 *	<0.0001 *	0.73	0.78	0.46	0.65	0.66	<0.0001 *	0.52	0.84

Where Y_j_ represents the predicted response (dependent variables), the model intercept (β_0_), linear terms (β_1_, β_2_, and β_4_), interaction terms (β_11_, β_22_, and β_44_) and quadratic terms (β_12_, β_14_, and β_24_), and X_1_ (Sample (g) solvent (g)^−1^), X_2_ (Extraction time (min)), and X_4_ (Extraction temperature (°C)) are the independent variables.* Independent effects are statistically significant if *p* < 0.05.

**Table 6:** Analysis of variance (ANOVA) of responses for ultrasound- assisted and microwave-assisted extractions.

Response	Source	df	SS	MS	F Ratio	Prob > F	Lack-of-Fit	R^2^	Adj. R^2^
Ultrasound-assisted extraction using model B (sample (g) solvent (g)^−1^, duty cycle (%), and extraction temperature (°C) as independent parameters)									
THC	Model	9	1.57	0.17	5.57	0.001 *	0.79	0.70	0.58
	Error	21	0.66	0.03					
THCA	Model	9	750.49	83.39	2.32	0.05 (0.005 *)	0.77 (0.33)	0.51	0.28
	Error	21	755.83	35.99					
Total THC	Model	9	637.95	70.88	2.49	0.04 *	0.74	0.52	0.31
	Error	21	598.79	28.51					
CBG	Model	9	0.05	0.01	4.67	0.002 *	0.98	0.67	0.52
	Error	21	0.02	0.00					
CBGA	Model	9	0.36	0.04	5.68	0.001 *	0.82	0.71	0.58
	Error	21	0.15	0.01					
Total CBG	Model	9	0.53	0.06	5.81	0.0004 *	0.83	0.71	0.59
	Error	21	0.22	0.01					
THCVA	Model	9	4.23	0.47	4.10	0.004 *	0.89	0.64	0.48
	Error	21	2.41	0.11					
CBCA	Model	9	0.04	0.00	3.56	0.008 *	0.66	0.60	0.43
	Error	21	0.03	0.00					
Total terpenes	Model	9	1.13	0.13	1.68	0.16 (0.02 *)	0.18 (0.05)	0.62	0.47
	Error	21	1.56	0.07					
Yield	Model	9	582.84	64.76	1.52	0.20 (0.04 *)	0.37 (0.06)	0.69	0.44
	Error	21	893.61	42.55					
Microwave-assisted extraction using model F (sample (g) solvent (g)^−1^ and extraction temperature (°C) as independent parameters)									
THC	Model	5	1.16	0.23	5.28	0.01 *	0.22	0.65	0.53
	Error	14	0.62	0.04					
THCA	Model	5	310.11	62.02	4.99	0.01 *	0.1	0.64	0.51
	Error	14	174.18	12.44					
Total THC	Model	5	253.77	50.75	4.81	0.01 *	0.09	0.63	0.5
	Error	14	147.7	10.55					
CBG	Model	5	0.01	0	4.67	0.01 *	0.12	0.63	0.49
	Error	14	0	0					
CBGA	Model	5	0.07	0.01	4.59	0.01 *	0.08	0.62	0.49
	Error	14	0.04	0.00					
Total CBG	Model	5	0.1	0.02	4.91	0.01 *	0.11	0.64	0.51
	Error	14	0.06	0					
THCVA	Model	5	1.03	0.21	5.21	0.01 *	0.14	0.65	0.53
	Error	14	0.56	0.04					
CBCA	Model	5	0.12	0.02	4.67	0.01 *	0.15	0.63	0.49
	Error	14	0.07	0.01					
Total terpenes	Model	5	0.66	0.13	2.96	0.04 *	0.05	0.51	0.34
	Error	14	0.63	0.04					
Yield	Model	5	616.93	123.4	30.46	<0.001 *	<0.001 *	0.92	0.89
	Error	14	56.71	4.05					

Effects are statistically significant if *p* value * < 0.05. *p*-values for ANOVA and lack-of-fit for the revised model B, which has only sample (g) solvent (g)^−1^ and extraction temperature (°C) as independent parameters. Degree of freedom (df), Sum of squares (SS), and Mean square (MS).

**Table 7:** Optimal experimental conditions for ultrasound-assisted and microwave-assisted extraction systems and predicted response values.

Extraction Method	Ultrasound-Assisted Extraction	Microwave-Assisted Extraction
Desirability	0.83	0.75
Sample (g) solvent (40 mL)^−1^	2.1	2.19
Sample (g) solvent (g)^−1^	1/15	1/14.43
Duty Cycle (%)	80	NA
Extraction temperature (°C)	60	60
Extraction time (min)	30	30
**Concentration of cannabinoids and total terpenes (g 100 g dry matter^−1^)**		
THC	1.06	0.92
THCA	28.52	19.95
Total THC	26	18.42
CBG	0.18	0.1
CBGA	0.48	0.3
Total CBG	0.6	0.37
THCVA	1.86	1.13
CBCA	0.17	0.39
Total terpenes	1.2	1.03
Extraction yield	29.81	25.52

NA: not applicable.

**Table 8:** Dilution factors used for cannabinoid and terpene analyses of cannabis biomass used for extraction.

Dilution Factor	Approximate Initial Mass of Biomass (g)
Cannabinoid analysis	
5000-fold	10
3000-fold	6
1500-fold	3
1000-fold	2
Terpene analysis	
1000-fold	10
500-fold	6
200-fold	3
100-fold	2

**Table 9:** Gas chromatography oven temperature program.

Retention Time (min)	Rate (°C min^−1^)	Target Value (°C)	Hold Time (min)
2.0	0.0	65.0	2.0
8.0	10.0	125.0	0.0
18.3	15.0	250.0	2.0
25.0	30.0	300.0	5.0
25.0	Stop Time		

**Table 10:** Uncoded and coded levels of the independent variables for ultrasound- and microwave-assisted extraction of cannabis.

Ultrasound-Assisted Extraction						
Independent variables	Symbol	Coded levels				
		−2	−1	0	+1	+2
Sample (g) solvent (mL)^−1^	X_1_	0/40	6.31/40	3.16/40	2.1/40	1.58/40
Sample (g) solvent (g)^−1^	X_1_	1/0	1/5	1/10	1/15	1/20
Extraction time (min)	X_2_	0	10	20	30	40
Duty cycle (%)	X_3_	20	40	60	80	100
Extraction temperature (°C)	X_4_	30	40	50	60	70
**Microwave-assisted extraction**						
Independent variables	Symbol	Coded levels				
		−1.682	−1	0	+1	+1.682
Sample (g) solvent (mL)^−1^	X_1_	19.84/40	6.31/40	3.16/40	2.1/40	1.71/40
Sample (g) solvent (g)^−1^	X_1_	1/1.59	1/5	1/10	1/15	1/18.41
Extraction time (min)	X_2_	3.18	10	20	30	36.82
Extraction temperature (°C)	X_4_	33.18	40	50	60	66.82

**Table 11:** Multiple regression equation for ultrasound-assisted extraction and microwave-assisted extraction.

Model	Multiple Regression Equation	Equation No.
Ultrasound-assisted extraction		
Model A	Y_j_ = β_0_ + β_1_X_1_ + β_2_X_2_ + β_3_X_3_ + β_4_X_4_ + β_11_X_1_X_1_ + β_22_X_2_X_2_ + β_33_X_3_X_3_ + β_44_X_4_X_4_ + β_12_X_1_X_2_ + β_13_X_1_X_3_ + β_14_X_1_X_4_ + β_23_X_2_X_3_ + β_24_X_2_X_4_ + β_34_X_3_X_4_	4
Model B	Y_j_ = β_0_ + β_1_X_1_ + β_3_X_3_ + β_4_X_4_ + β_11_X_1_X_1_ + β_33_X_3_X_3_ + β_44_X_4_X_4_ + β_13_X_1_X_3_ + β_14_X_1_X_4_ + β_34_X_3_X_4_	5
Model C	Y_j_ = β_0_ + β_1_X_1_ + β_2_X_2_ + β_4_X_4_ + β_11_X_1_X_1_ + β_22_X_2_X_2_ + β_44_X_4_X_4_ + β_12_X_1_X_2_ + β_14_X_1_X_4_ + β_24_X_2_X_4_	6
Model D	Y_j_ = β_0_ + β_1_X_1_ + β_2_X_2_ + β_3_X_3_ + β_11_X_1_X_1_ + β_22_X_2_X_2_ + β_33_X_3_X_3_ + β_12_X_1_X_2_ + β_13_X_1_X_3_ + β_23_X_2_X_3_	7
Microwave-assisted extraction		
Model E	Y_j_ = β_0_ + β_1_X_1_ + β_2_X_2_ + β_4_X_4_ + β_11_X_1_X_1_ + β_22_X_2_X_2_ + β_44_X_4_X_4_ + β_12_X_1_X_2_ + β_14_X_1_X_4_ + β_24_X_2_X_4_	8
Model F	Y_j_ = β_0_ + β_1_X_1_ + β_4_X_4_ + β_11_X_1_X_1_ + β_44_X_4_X_4_ + β_14_X_1_X_4_	9
Model G	Y_j_ = β_0_ + β_1_X_1_ + β_2_X_2_ + β_11_X_1_X_1_ + β_22_X_2_X_2_ + β_12_X_1_X_2_	10

Where Y_j_ represents the predicted response (dependent variables), the model intercept (β_0_), linear terms (β_1_, β_2_, β_3_, and β_4_), interaction terms (β_11_, β_22_, β_33_, and β_44_) and quadratic terms (β_12_, β_13_, β_14_, β_23_, β_24_, and β_34_), and X_1_ (Sample (g) solvent (g)^−1^), X_2_ (Extraction time (min)), X_3_ (Duty cycle (%)), and X_4_ (Extraction temperature (°C)) are the independent variables.

## Data Availability

Not applicable.

## Supplementary Materials

The following supporting information can be downloaded at: https://www.mdpi.com/article/10.3390/molecules27248803/s1, Table S1: Ultrasound-assisted extraction of cannabis using model B (sample (g) solvent (g)^−1^, duty cycle (%), and extraction temperature (°C) as independent parameters). Table S2: Ultrasound-assisted extraction of cannabis using model C (sample (g) solvent (g)^−1^, extraction time (min), and extraction temperature (°C) as independent parameters). Table S3: Ultrasound-assisted extraction of cannabis using model D (sample (g) solvent (g)^−1^, extraction time (min), and duty cycle (%) as independent parameters). Table S4: Microwave-assisted extraction of cannabis using model F (sample (g) solvent (g)^−1^ and extraction temperature (°C) as independent parameters). Table S5: Microwave-assisted extraction of cannabis using model G (sample (g) solvent (g)^−1^ and extraction time (min) as independent parameters).
