# Supplementary material for: Microwave- and Ultrasound-Assisted Extraction of Cannabinoids and Terpenes from Cannabis Using Response Surface Methodology
Source: Molecules. 2022 Dec 12;27(24):8803. doi: 10.3390/molecules27248803 (PMC9784742; doi:10.3390/molecules27248803)
Supplement: Supplementary file 1 [file molecules-27-08803-s001.zip › molecules-2035534-supplementary.pdf]

Table S1. Ultrasound-assisted extraction of cannabis using model B (sample (g) solvent (g)$^{-1}$, duty cycle (%), and extraction temperature (°C) as independent parameters)

**Response/dependent variables**		**Regression model effect parameters**									
		Intercept	Linear			Interaction			Quadratic		
		β_0	β_1	β_3	β_4	β_{13}	β_{14}	β_{34}	β_{11}	β_{33}	β_{44}
THC	Coefficient	0.69	0.25	0.05	0.02	0.01	0.001	-0.002	-0.005	0.02	0.03
	p value	<.0001*	<.0001*	0.2	0.53	0.83	0.99	0.97	0.88	0.52	0.37
THCA	Coefficient	23.68	4.82	0.51	0.28	-0.52	-0.25	-0.1	-1.9	0.95	1.03
	p value	<.0001*	0.001*	0.68	0.82	0.73	0.87	0.95	0.1	0.41	0.36
Total THC	Coefficient	20.76	4.48	0.5	0.27	-0.44	-0.22	-0.1	-1.46	1.06	1.15
	p value	<.0001*	0.0005*	0.65	0.81	0.74	0.87	0.94	0.16	0.3	0.26
CBG	Coefficient	0.14	0.04	0.01	0.003	-0.003	-0.002	0.001	-0.01	0.003	0.004
	p value	<.0001*	<.0001*	0.37	0.67	0.71	0.82	0.94	0.2	0.64	0.5
CBGA	Coefficient	0.34	0.12	0.01	0.001	-0.003	0.002	0.01	-0.01	-0.002	0.01
	p value	<.0001*	<.0001*	0.52	0.94	0.88	0.93	0.66	0.54	0.89	0.42
Total CBG	Coefficient	0.41	0.15	0.02	0.01	-0.01	0.003	0.01	-0.01	0.01	0.02
	p value	<.0001*	<.0001*	0.46	0.80	0.83	0.9	0.75	0.63	0.66	0.25
THCVA	Coefficient	1.32	0.4	0.06	0.03	-0.01	0.002	-0.001	-0.06	0.05	0.06
	p value	<.0001*	<.0001*	0.39	0.62	0.87	0.98	0.99	0.39	0.42	0.34
CBCA	Coefficient	0.12	0.04	-0.002	0.01	-0.004	0.01	-0.004	-0.002	0.004	0.01
	p value	<.0001*	<.0001*	0.78	0.47	0.63	0.37	0.63	0.72	0.57	0.27
Total terpenes	Coefficient	0.95	0.2	0.001	0.003	-0.02	-0.01	0.003	-0.02	0.04	0.05

	p value	<.0001*	0.002*	0.98	0.95	0.81	0.83	0.96	0.69	0.39	0.33
Yield	Coefficient	24.59	4.01	0.47	0.36	-0.29	-0.12	-0.1	-1.64	1.21	1.32
	p value	<.0001*	0.0067*	0.73	0.79	0.86	0.94	0.95	0.19	0.33	0.29

Table S2. Ultrasound-assisted extraction of cannabis using model C (sample (g) solvent (g) $^{-1}$, extraction time (min), and extraction temperature ($^{\circ}$C) as independent parameters).

**Response/dependent variables**		**Regression model effect parameters**									
		Intercept	Linear			Interaction			Quadratic		
		β_0	β_1	β_2	β_4	β_{12}	β_{14}	β_{24}	β_{11}	β_{22}	β_{44}
THC	Coefficient	0.77	0.25	0.10	0.02	0.02	0.001	-0.001	-0.01	-0.07	0.02
	p value	<.0001*	<.0001*	0.002*	0.41	0.64	0.99	0.99	0.59	0.01*	0.39
THCA	Coefficient	26.98	4.82	2.99	0.28	-0.94	-0.25	0.02	-2.24	-2.63	0.69
	p value	<.0001*	<.0001*	0.002*	0.74	0.37	0.82	0.99	0.01*	0.01*	0.38
Total THC	Coefficient	23.73	4.48	2.72	0.27	-0.82	-0.22	0.01	-1.77	-2.16	0.84
	p value	<.0001*	<.0001*	0.003*	0.73	0.41	0.83	0.99	0.02*	0.01*	0.26
CBG	Coefficient	0.15	0.04	0.02	0.002	-0.002	-0.002	0.002	-0.01	-0.02	0.002
	p value	<.0001*	<.0001*	0.001*	0.49	0.71	0.71	0.71	0.01*	0.01*	0.53
CBGA	Coefficient	0.37	0.12	0.03	0.001	-0.003	0.002	-0.01	-0.01	-0.04	0.01
	p value	<.0001*	<.0001*	0.03*	0.93	0.85	0.91	0.62	0.29	0.01*	0.44
Total CBG	Coefficient	0.46	0.15	0.04	0.01	-0.01	0.003	-0.004	-0.01	-0.04	0.02
	p value	<.0001*	<.0001*	0.01*	0.74	0.78	0.88	0.83	0.35	0.01*	0.25
THCVA	Coefficient	1.48	0.4	0.21	0.03	-0.06	0.002	-0.001	-0.07	-0.13	0.04
	p value	<.0001*	<.0001*	0.001*	0.45	0.32	0.97	0.99	0.09	0.01*	0.29
CBCA	Coefficient	0.14	0.04	0.02	0.01	-0.003	0.01	-0.01	0.004	-0.01	0.01
	p value	<.0001*	<.0001*	0.01*	0.36	0.66	0.26	0.44	0.45	0.02*	0.27
Total terpenes	Coefficient	1.08	0.20	0.08	0.003	-0.01	-0.01	0.02	-0.03	-0.1	0.04

	p value	<.0001*	0.001*	0.13	0.94	0.91	0.81	0.76	0.45	0.04*	0.41
Yield	Coefficient	27.99	4.01	2.64	0.36	-0.21	-0.12	-0.12	-2	-2.47	0.97
	p value	<.0001*	0.001*	0.02*	0.75	0.88	0.93	0.93	0.06	0.02*	0.34

Effects are statistically significant if *p value* * < 0.05

Table S3. Ultrasound-assisted extraction of cannabis using model D (sample (g) solvent (g) $^{-1}$, extraction time (min), and duty cycle (%) as independent parameters).

**Response/dependent variables**		**Regression model effect parameters**									
		Intercept	Linear			Interaction			Quadratic		
		β_0	β_1	β_2	β_3	β_{12}	β_{13}	β_{23}	β_{11}	β_{22}	β_{33}
THC	Coefficient	0.78	0.25	0.1	0.05	0.02	0.01	0.01	-0.01	-0.07	0.01
	p value	<.0001*	<.0001*	0.001*	0.08	0.62	0.77	0.8	0.55	0.01*	0.62
THCA	Coefficient	27.07	4.82	2.99	0.51	-0.95	-0.52	0.11	-2.25	-2.64	0.6
	p value	<.0001*	<.0001*	0.002*	0.55	0.37	0.62	0.91	0.01*	0.01*	0.45
Total THC	Coefficient	23.82	4.48	2.72	0.5	-0.82	-0.44	0.11	-1.78	-2.17	0.74
	p value	<.0001*	<.0001*	0.002*	0.54	0.41	0.65	0.91	0.02*	0.01*	0.31
CBG	Coefficient	0.16	0.04	0.02	0.01	0.001	-0.002	-0.003	-0.01	-0.02	0.001
	p value	<.0001*	<.0001*	0.001*	0.13	0.7	0.53	0.9	0.01*	0.02*	0.78
CBGA	Coefficient	0.39	0.12	0.03	0.01	-0.003	-0.003	-0.003	-0.01	-0.04	-0.01
	p value	<.0001*	<.0001*	0.03*	0.4	0.85	0.85	0.85	0.24	0.01*	0.56
Total CBG	Coefficient	0.47	0.15	0.04	0.02	-0.01	-0.01	-0.003	-0.02	-0.04	0.002
	p value	<.0001*	<.0001*	0.01*	0.35	0.78	0.78	0.88	0.31	0.01*	0.88
THCVA	Coefficient	1.49	0.4	0.21	0.06	-0.07	-0.01	-0.002	-0.07	-0.12	0.03
	p value	<.0001*	<.0001*	0.01*	0.19	0.31	0.79	0.97	0.08	0.01*	0.41
CBCA	Coefficient	0.14	0.04	0.02	-0.002	-0.003	-0.004	0.004	-0.004	-0.01	0.002
	p value	<.0001*	<.0001*	0.02*	0.74	0.68	0.57	0.57	0.44	0.03*	0.75
Total terpenes	Coefficient	1.08	0.2	0.08	0.001	-0.01	-0.02	0.02	-0.03	-0.1	0.03

	p value	<.0001*	0.0005*	0.13	0.98	0.91	0.78	0.8	0.45	0.04*	0.51
Yield	Coefficient	28.1	4.01	2.64	0.47	-0.21	-0.29	-0.18	-2.01	-2.49	0.85
	p value	<.0001*	0.0014*	0.02*	0.67	0.88	0.83	0.89	0.06	0.02*	0.4

Effects are statistically significant if *p value* * < 0.05

Table S4. Microwave-assisted extraction of cannabis using model F (sample (g) solvent (g) $^{-1}$ and extraction temperature (˚C) as independent parameters).

**Response/dependent variables**		**Regression model effect parameters**					
		Intercept	Linear		Interaction	Quadratic	
		β_0	β_1	β_4	β_{14}	β_{11}	β_{44}
THC	Coefficient	0.63	0.05	0.25	-0.01	-0.11	0.09
	p value	<.0001*	0.37	0.001*	0.89	0.07	0.14
THCA	Coefficient	17.18	4.24	0.35	0.02	-2.03	0.24
	p value	<.0001*	0.001*	0.72	0.98	0.04*	0.8
Total THC	Coefficient	15.7	3.78	0.55	0.01	-1.89	0.3
	p value	<.0001*	0.001*	0.54	0.96	0.04*	0.73
CBG	Coefficient	0.09	0.02	0.01	-0.001	-0.01	0.002
	p value	<.0001*	0.001*	0.32	0.85	0.06	0.61
CBGA	Coefficient	0.25	0.06	0.01	0.01	-0.03	0.01
	p value	<.0001*	0.001*	0.58	0.8	0.06	0.66
Total CBG	Coefficient	0.3	0.08	0.01	0.004	-0.04	0.01
	p value	<.0001*	0.001*	0.46	0.87	0.05	0.63
THCVA	Coefficient	0.96	0.25	0.02	0.01	-0.11	0.01
	p value	<.0001*	0.0004*	0.67	0.92	0.06	0.87
CBCA	Coefficient	0.34	0.08	0.01	0.001	-0.04	0.002
	p value	<.0001*	0.001*	0.69	0.97	0.05	0.9
Total terpenes	Coefficient	0.93	0.16	0.03	0.07	-0.14	-0.02

	p value	<.0001*	0.02*	0.67	0.38	0.03*	0.77
Yield	Coefficient	24.24	5.32	0.17	-0.37	-3.96	-0.16
	p value	<.0001*	<.0001*	0.76	0.61	<.0001*	0.76

Effects are statistically significant if *p value* * < 0.05

Table S5. Microwave-assisted extraction of cannabis using model G (sample (g) solvent (g) $^{-1}$ and extraction time (min) as independent parameters).

**Response/dependent variables**		**Regression model effect parameters**					
		Intercept	Linear		Interaction	Quadratic	
		β_0	β_1	β_2	β_{12}	β_{11}	β_{22}
THC	Coefficient	0.67	0.05	0.03	0.14	-0.11	0.04
	p value	<.0001*	0.54	0.71	0.22	0.19	0.67
THCA	Coefficient	17.26	4.24	0.64	2.12	-2.04	0.14
	p value	<.0001*	0.0002*	0.46	0.07	0.03*	0.86
Total THC	Coefficient	15.81	3.78	0.59	2	-1.9	0.16
	p value	<.0001*	0.0003*	0.46	0.07	0.02*	0.84
CBG	Coefficient	0.09	0.02	0.003	0.01	-0.01	0.003
	p value	<.0001*	0.0004*	0.43	0.07	0.03*	0.57
CBGA	Coefficient	0.25	0.06	0.01	0.03	-0.03	0.004
	p value	<.0001*	0.0003*	0.33	0.07	0.03*	0.72
Total CBG	Coefficient	0.31	0.08	0.01	0.04	-0.04	0.004
	p value	<.0001*	0.0002*	0.38	0.06	0.03*	0.77
THCVA	Coefficient	0.96	0.25	0.05	0.12	-0.11	0.01
	p value	<.0001*	0.0001*	0.32	0.08	0.03*	0.86
CBCA	Coefficient	0.34	0.08	0.01	0.05	-0.04	0.004
	p value	<.0001*	0.0002*	0.4	0.06	0.03*	0.8
Total terpenes	Coefficient	0.92	0.16	0.05	0.1	-0.14	-0.001

	p value	<.0001*	0.01*	0.38	0.18	0.02*	0.98
Yield	Coefficient	23.81	5.32	-0.21	0.61	-3.91	0.4
	p value	<.0001*	<.0001*	0.69	0.39	<.0001*	0.44
